# Supplementary material for: A cellular basis for mapping behavioural structure
Source: Nature. 2024 Nov 6;636(8043):671–80. doi: 10.1038/s41586-024-08145-x (PMC11655361; doi:10.1038/s41586-024-08145-x)
Supplement: Supplementary file 1 — This table outlines the numbers of mice, recording days, tasks, sessions, neurons and neuron pairs (as appropriate) for each analysis and the criteria used for inclusion. [file 41586_2024_8145_MOESM1_ESM.pdf]

nature portfolio

https://doi.org/10.1038/s41586-024-08145-x

Supplementary information

A cellular basis for mapping behavioural structure

In the format provided by the authors and unedited

Supplementary Table 1 - Break down of sample numbers in data figures

Figure	Criteria	Mice	Recording Days	Neurons/Neuron pairs
**Figure 1**<br>1c-e<br>Extended data 1b, d(left),e,h-k,n-q)	-All mice	13 (400 tasks)	NA	NA
Extended data 1c (middle,right)	-All neuronal mice (mice form which neuronal data was recorded)	7 (280 tasks)		
Extended data 1f,g	-All mice that experienced additional ABCD tasks with tone omitted from 50% of trials	3 (26 tasks)		
Extended data 1l,m	-All mice that experienced ABCDE tasks	2 (24 tasks)		
**Figure 2**<br>2f	-All recorded neurons on days when animal had a repeat session of task X	7	44 days	2461 neurons
2g<br>Extended data 2d,e,g	-Concatenated double-days	7	25 days	1252 neurons
Extended data 2f	-Concatenated double days<br>-Only goal-progress neurons<br>-Only neurons with at least one significant goal-progress peak	7	25 days	873 neurons
2h,i	-Concatenated double-days<br>-10 or more simultaneously recorded neurons<br>-At least 6 unique tasks	7	20 days	1170 neurons
Extended data 2i	-Neuropixels recordings	2	12 days	446 neurons
Extended data 2j,k	-Concatenated double-days<br>-10 or more simultaneously recorded neurons<br>with no significant spatial tuning in GLM<br>-At least 6 unique tasks	3	8 days	184 neurons
Extended data 3b	-Days with ABCDE tasks<br>-Concatenated double days<br>-State-tuned neurons	2	4 days	189 neurons
Extended data 3d-f	-Days with ABCD and ABCDE tasks<br>-Concatenated double days	2	2 days	111 neurons
Extended data 3g,h	-Days with ABCDE tasks<br>-Concatenated double days<br>-10 or more simultaneously recorded neurons	2	4 days	288 neurons

**Figure 3**<br>3b	-3 unique tasks<br>-state-tuned neurons	7	X vs Y/Z<br>50 days<br>X vs X'<br>37 days	X vs Y/Z<br>1594 neurons<br>X vs X'<br>1160 neurons
Extended data figure 4b	-3 unique tasks<br>-state-tuned neurons with state-tuning coefficient higher than 99th percentile of permuted distribution	7	X vs Y/Z<br>46 days<br>X vs X'<br>35 days	X vs Y/Z<br>1061 neurons<br>X vs X'<br>770 neurons
Extended data figure 4c	-3 unique tasks<br>-state-tuned neurons<br>-only neurons with no discrepancy between "peak-to-peak" angles and "best-rotation" angles	7	X vs Y/Z<br>41 days<br>X vs X'<br>31 days	X vs Y/Z<br>369 neurons<br>X vs X'<br>240 neurons
Extended data figure 4d	-3 unique tasks<br>-only neurons with no significant spatial tuning in GLM	7	X vs Y/Z<br>47 days<br>X vs X'<br>36 days	X vs Y/Z<br>704 neurons<br>X vs X'<br>507 neurons
3c; Extended data figure 4h	-3 unique tasks<br>-state-tuned neurons<br>-10 or more simultaneously recorded neurons	7	X vs Y/Z<br>38 days<br>X vs X'<br>34 days	X vs Y/Z:<br>35164 pairs<br>X vs X'<br>23674 pairs
Extended data figure 4e	-3 unique tasks<br>-state-tuned neurons with state-tuning coefficient higher than 99th percentile of permuted distribution<br>-10 or more simultaneously recorded neurons	7	X vs Y/Z<br>34 days<br>X vs X'<br>30 days	X vs Y/Z<br>17671 pairs<br>X vs X'<br>11716 pairs
Extended data figure 4f	-3 unique tasks<br>-state-tuned neurons<br>-10 or more simultaneously recorded neurons<br>-only neurons with no discrepancy between "peak-to-peak" angles and "best-rotation" angles	4	X vs Y/Z<br>16 days<br>X vs X'<br>12 days	X vs Y/Z:<br>1642 pairs<br>X vs X'<br>657 pairs
Extended data figure 4g	-3 unique tasks<br>-10 or more simultaneously recorded neurons<br>with no significant spatial tuning in GLM	4	X vs Y/Z<br>13 days<br>X vs X'<br>12 days	X vs Y/Z:<br>6996 pairs<br>X vs X'<br>4822 pairs
Extended data figure 4i	-Concatenated double days (6 unique tasks)<br>-state tuned neurons<br>-10 or more simultaneously recorded neurons<br>-more than one site along AP axis (cambridge neurotech probes)<br>-At least 2 pairs coherent along all 6 tasks	4	11 days	3567 pairs
3d	-3 unique tasks<br>-state-tuned neurons<br>-10 or more simultaneously recorded neurons	7	38 days	1434 neurons
Extended data figure 4j	-3 unique tasks<br>-state-tuned neurons	2	12 days	576 neurons

Extended data figure 4k	-Neuropixels recordings<br><br>-3 unique tasks<br>-state-tuned neurons<br>-Neuropixels recordings<br>-10 or more simultaneously recorded neurons within the same DV bin	2	10 days	5210 pairs<br><br>*Note that on all included days animals completed 3 unique tasks. On the majority, but not all, of those days animals additionally had a repeat session of task X' at the end of the recording day.
**Figure 4**<br>Extended data figure 5	NA	NA	NA	NA
**Figure 5**<br>5b,c, Extended data figure 7a,b	-concatenated double-days<br>-All state tuned neurons	7	25	All state-tuned<br>737 neurons<br><br>Non-zero lag state tuned<br>544 neurons*<br><br>-Distal lag (> 1 state either side of anchor)<br>Non-zero lag state tuned<br>305 neurons*
Extended data figure 7c,d	-concatenated double-days<br>-All consistently anchored neurons (neurons with the same anchor in >50% of tasks)	7	25	672 neurons
Extended data figure 7f	-concatenated double-days<br>-Neuropixels recordings<br>-Consistently anchored neurons	2	6	247 neurons
Extended data figure 7g	-concatenated double-days<br>-ABCDE days<br>-All state-tuned neurons	2	4	All state-tuned<br>188 neurons<br><br>Non-zero lag state tuned<br>153 neurons*<br><br>-Distal lag (> 1 state either side of anchor)<br>Non-zero lag state tuned<br>31 neurons*
5f, Extended data figure 7h,i	-concatenated double-days<br>-All state tuned neurons	7	25	All state-tuned<br>738 neurons<br><br>Non-zero lag state tuned<br>285 neurons*<br><br>-Distal peak spatial correlation (> 1 state either side of anchor)<br>Non-zero lag state tuned<br>135 neurons*

5h, Extended data figure 8a,e	-concatenated double-days<br>-State tuned neurons<br>-non-zero regression coefficients	7	25	All state-tuned<br>489 neurons<br><br>Non-zero lag state tuned<br>329 neurons*<br><br>-Distal peak coefficient (> 1 state either side of anchor) Non-zero lag state tuned<br>224 neurons*
Extended data figure 8b	-concatenated double-days<br>-All state tuned neurons with state-tuning coefficient higher than 99th percentile of permuted distribution<br>-non-zero regression coefficients	7	25	All state-tuned<br>349 neurons<br><br>Non-zero lag state tuned<br>227 neurons*<br><br>-Distal peak coefficient (> 1 state either side of anchor) Non-zero lag state tuned<br>154 neurons*
Extended data figure 8c	-concatenated double-days<br>-State tuned neurons<br>-No trace of tuning to current trajectory<br>-non-zero regression coefficients	7	25	112 neurons
Extended data figure 8d	-concatenated double-days<br>-State tuned neurons<br>-non-zero regression coefficients (Poisson GLM)	7	25	All state-tuned<br>489 neurons<br><br>Non-zero lag state tuned<br>346 neurons*<br><br>-Distal peak coefficient (> 1 state either side of anchor) Non-zero lag state tuned<br>229 neurons*<br><br>*Note: lag from anchor is calculated separately for each analysis method - hence numbers of non-zero lag neurons are different for each method
**Figure 6**	All panels :<br>-concatenated double days<br>-at least 20 trials to a given anchor (location/goal-progress conjunction)<br>-Only non-zero lag neurons (lag of > 30 degrees in task space either side of anchor)<br>-Consistently anchored neurons (neurons with the same anchor in >50% of tasks) - unless otherwise stated<br>-Firing rate bar-graphs: at least one instance of both anchor visit combinations (e.g. 0:0 and 0:1)			

	In addition to the above conditions:			
Extended data 9b	-All consistently anchored neurons	7	23	355 neurons<br>(0:0,0:1) 131 tasks<br>(1:0,1:1) 123 tasks
6c, Extended data 9c	-All consistently anchored neurons	7	23	355 neurons<br>131 tasks
Extended data 9d	-Only distal non-zero lag neurons (lag of > 1 state either side of anchor)	7	23	200 neurons<br>(0:0,0:1) 128 tasks<br>(1:0,1:1) 115 tasks
Extended data 9e	-Only neurons anchored to non-rewarded locations<br>-tasks as Ns	7	22	234 neurons<br>(0:0,0:1) 126 tasks<br>(1:0,1:1) 120 tasks
Extended data 9f	-Only neurons anchored to non-rewarded locations<br>-anchorsxtasks as Ns	7	22	234 neurons<br>(0:0,0:1) 821 anchor-tasks<br>(1:0,1:1) 529 anchor-tasks
Extended data 9g	-Only neurons anchored to non-rewarded locations - even if not consistently anchored<br>-Tasks as Ns	7	23	504 neurons<br>(0:0,0:1)<br>130 tasks<br>(1:0,1:1)<br>130 tasks
Extended data 9h	-Only neurons anchored to non-rewarded locations - even if not consistently anchored<br>-anchorsxtasks as Ns	7	23	504 neurons<br>(0:0,0:1)<br>1273 anchor-tasks<br>(1:0,1:1)<br>826 anchor-tasks
Extended data 9i	-ABCDE days	2	4	110 neurons<br>(0:0,0:1)<br>24 tasks<br>(1:0,1:1)<br>24 tasks
**Figure 7**	All panels:<br>-concatenated double days<br>-All consistently anchored neurons (neurons with the same anchor in >50% of tasks)<br><br>In addition to the above conditions:			
7c	-All pairs either sharing the same anchor or across anchors	7	24	430 pairs (within)<br>13932 pairs (between)
7d , Extended data 10a	-All pairs sharing the same anchor	7	24	430 pairs
7e	-All recording days with more than 10 co-anchored pairs in each of the 0-180 and 180-360 forward distance brackets	5	10	59 sleep sessions
Extended data 10b,c	-All pairs sharing the same anchor<br>-session type (pre or post sleep) experienced on a given day	7	24	512 pairs (pre-task)<br>429 pairs (post-task)

				*pair numbers are averaged across all sleep sessions - hence when sessions are broken down (e.g. to pre and post sleep) the numbers are not exactly equal since not all sleep sessions were sampled on all recording days
