# Supplementary material for: A cellular basis for mapping behavioural structure
Source: Nature. 2024 Nov 6;636(8043):671–80. doi: 10.1038/s41586-024-08145-x (PMC11655361; doi:10.1038/s41586-024-08145-x)
Supplement: Supplementary file 2 — Reporting Summary [file 41586_2024_8145_MOESM2_ESM.pdf]

nature portfolio

Corresponding author(s): Mohamady El-Gaby
Tim Behrens

Last updated by author(s): 15/08/24

Reporting Summary

Nature Portfolio wishes to improve the reproducibility of the work that we publish. This form provides structure for consistency and transparency in reporting. For further information on Nature Portfolio policies, see our Editorial Policies and the Editorial Policy Checklist.

Statistics

For all statistical analyses, confirm that the following items are present in the figure legend, table legend, main text, or Methods section.

n/a	Confirmed	
☐	☒	The exact sample size (*n*) for each experimental group/condition, given as a discrete number and unit of measurement
☐	☒	A statement on whether measurements were taken from distinct samples or whether the same sample was measured repeatedly
☐	☒	The statistical test(s) used AND whether they are one- or two-sided<br>*Only common tests should be described solely by name; describe more complex techniques in the Methods section.*
☐	☒	A description of all covariates tested
☐	☒	A description of any assumptions or corrections, such as tests of normality and adjustment for multiple comparisons
☐	☒	A full description of the statistical parameters including central tendency (e.g. means) or other basic estimates (e.g. regression coefficient) AND variation (e.g. standard deviation) or associated estimates of uncertainty (e.g. confidence intervals)
☐	☒	For null hypothesis testing, the test statistic (e.g. *F*, *t*, *r*) with confidence intervals, effect sizes, degrees of freedom and *P* value noted<br>*Give P values as exact values whenever suitable.*
☒	☐	For Bayesian analysis, information on the choice of priors and Markov chain Monte Carlo settings
☒	☐	For hierarchical and complex designs, identification of the appropriate level for tests and full reporting of outcomes
☐	☒	Estimates of effect sizes (e.g. Cohen's *d*, Pearson's *r*), indicating how they were calculated

Our web collection on statistics for biologists contains articles on many of the points above.

Software and code

Policy information about availability of computer code

Data collection	Open source pyControl v1.2 (behaviour) and OpenEphys v0.4.3 (electrophysiology) software was used for data collection
Data analysis	Preprocessing was carried out using Kilosort (versions 2.5 and 3) for spike sorting and DeepLabCut (2.0) a Python package for marker-less pose estimation based in the TensorFlow machine learning library for tracking animals in videos. HERBS software (0.2.8) was used to localise the silicon probes to anatomical locations on the mouse brain. All data was analysed using Python (3) code. This used custom-made code but made use of libraries such as numpy (1.22.0), scipy (1.10.1), matplotlib (3.7.3), sciKit learn (1.3.2), pandas (2.0.3) and seaborn (0.13.2).<br>Code used in this study is publicly available here: https://github.com/mohamadyelgaby/mFC_schema

For manuscripts utilizing custom algorithms or software that are central to the research but not yet described in published literature, software must be made available to editors and reviewers. We strongly encourage code deposition in a community repository (e.g. GitHub). See the Nature Portfolio guidelines for submitting code & software for further information.

Data

Policy information about availability of data

All manuscripts must include a data availability statement. This statement should provide the following information, where applicable:

- Accession codes, unique identifiers, or web links for publicly available datasets
- A description of any restrictions on data availability
- For clinical datasets or third party data, please ensure that the statement adheres to our policy

Data used in this study is publicly available here (via OSF): https://doi.org/10.17605/OSF.IO/3D9R2
Details of the design and material of all maze components can be found in this open source link:
https://github.com/pyControl/hardware/tree/master/GridMaze
The mouse brain atlas (http://labs.gaidi.ca/mouse-brain-atlas/) was used for checking implantation coordinates.

Research involving human participants, their data, or biological material

Policy information about studies with human participants or human data. See also policy information about sex, gender (identity/presentation), and sexual orientation and race, ethnicity and racism.

Reporting on sex and gender	NA
Reporting on race, ethnicity, or other socially relevant groupings	NA
Population characteristics	NA
Recruitment	NA
Ethics oversight	NA

Note that full information on the approval of the study protocol must also be provided in the manuscript.

Field-specific reporting

Please select the one below that is the best fit for your research. If you are not sure, read the appropriate sections before making your selection.

☒ Life sciences ☐ Behavioural & social sciences ☐ Ecological, evolutionary & environmental sciences

For a reference copy of the document with all sections, see nature.com/documents/nr-reporting-summary-flat.pdf

Life sciences study design

All studies must disclose on these points even when the disclosure is negative.

Sample size	No statistical methods were used to pre-determine sample sizes but our sample sizes are similar to those reported in previous publications. e.g. Zhou et al 2021 (https://doi.org/10.1038/s41586-020-03061-2)
Data exclusions	Of the 13 animals, 7 animals in total were used for electrophysiological recordings, the remaining 6 animals are accounted for below:<br>● 3 animals (in cohort 1) were not implanted at any point<br>● 1 animal was implanted with silicon probes but was part of the first cohort so did not get to the 3 task days (i.e. only completed the first 10 tasks)<br>● 2 animals were implanted but their signal was lost before the 3 task days
Replication	All Behavioural and neuronal results were replicated across 4 cohorts of 13 mice.
Randomization	The sequences of tasks that the animals experienced throughout the experiment were randomised and counterbalanced.
Blinding	Only one experimental group (silicon probe implant in the frontal cortex) was used in this study and so blinding was not necessary.

Reporting for specific materials, systems and methods

We require information from authors about some types of materials, experimental systems and methods used in many studies. Here, indicate whether each material, system or method listed is relevant to your study. If you are not sure if a list item applies to your research, read the appropriate section before selecting a response.

Materials & experimental systems

n/a	Involved in the study
☒	☐ Antibodies
☒	☐ Eukaryotic cell lines
☒	☐ Palaeontology and archaeology
☐	☒ Animals and other organisms
☒	☐ Clinical data
☒	☐ Dual use research of concern
☒	☐ Plants

Methods

n/a	Involved in the study
☒	☐ ChIP-seq
☒	☐ Flow cytometry
☒	☐ MRI-based neuroimaging

Animals and other research organisms

Policy information about studies involving animals; ARRIVE guidelines recommended for reporting animal research, and Sex and Gender in Research

Laboratory animals	13 male C57BL/6 mice (Charles River Laboratories) aged 2-5 months at the time of testing. Animals were housed on a 12 hour light/dark cycle (7am-7pm) at 20-24°c and humidity of 55±10%.
Wild animals	No wild animals were used in the study.
Reporting on sex	Only male animals were used in the study as the weight of the implant used for recording precluded using females.
Field-collected samples	No field corrected samples were used in the study.
Ethics oversight	Experiments were carried out in accordance with Oxford University animal use guidelines and performed under UK Home Office Project Licence P6F11BC25.

Note that full information on the approval of the study protocol must also be provided in the manuscript.

Plants

Seed stocks	NA
Novel plant genotypes	NA
Authentication	NA
